# Second-Trimester Uterine Rupture After Conservative Myomectomy: A Case Report and Institutional Perspective

**DOI:** 10.7759/cureus.107129

**Published:** 2026-04-15

**Authors:** Platon Machavariani, Nickolas Kintraia, Natia Pkhaladze, Patman Tsaava, Ketevan Chichua

**Affiliations:** 1 Perinatology: Obstetrics and Gynecology, The First University Clinic of Tbilisi State Medical University (TSMU), Tbilisi, GEO; 2 Obstetrics and Gynecology, Tbilisi State Medical University (TSMU), Tbilisi, GEO; 3 Obstetrics and Gynecology, The First University Clinic of Tbilisi State Medical University (TSMU), Tbilisi, GEO; 4 Obstetrics and Gynecology, Chachava Clinic, Tbilisi, GEO

**Keywords:** emergency cesarean section, hemoperitoneum, high-risk pregnancy, leiomyoma surgery, myomectomy, obstetric hemorrhage, pregnancy complications, preterm delivery, uterine rupture, uterine scar integrity

## Abstract

Uterine rupture following myomectomy represents a rare but severe complication of pregnancy, with a reported incidence of less than 1%. It most commonly occurs during the late second or third trimester and is associated with significant maternal hemorrhage and fetal compromise.

We report the case of a 36-year-old G3P0 woman at 28 + 5 weeks of gestation with a history of prior laparoscopic myomectomy complicated by postoperative hemorrhage and sepsis, who presented with acute abdominal pain and hemodynamic instability. Emergency laparotomy revealed approximately 1000 mL hemoperitoneum and a 3 cm rupture of the posterior wall of the lower uterine segment. A live preterm neonate was delivered via cesarean section, followed by total hysterectomy due to extensive uterine pathology and ongoing hemorrhage. The postoperative course was uneventful, and the neonate required intensive care support. This case underscores the importance of maintaining a high index of suspicion for uterine rupture in pregnant patients with a history of uterine surgery and highlights the potential role of surgical technique and uterine wall reconstruction in determining subsequent scar integrity.

## Introduction

Uterine rupture is a rare but potentially catastrophic complication of pregnancy, particularly in women with a history of prior uterine surgery: myomectomy, C-section, metroplasty, and cervical cerclage [1-3]. Conservative myomectomy, performed to remove uterine leiomyomas while preserving fertility, has been associated with an estimated 0.5-1% risk of rupture in subsequent pregnancies, although the true incidence varies depending on surgical technique, fibroid characteristics, and individual patient factors [1,2]. Most ruptures occur in the late second or third trimester, often before the onset of labor, and may present with sudden abdominal pain, hemodynamic instability, intra-abdominal hemorrhage, or fetal compromise [3,4].

Several factors have been implicated in increasing the risk of post-myomectomy uterine rupture, including the number, size, and location of fibroids, the depth of myometrial incision, and the adequacy of uterine wall reconstruction, suturing techniques, interrupted sutures, individual stitches, or continuous sutures, suture layering: single-layer closure - one layer of sutures to approximate the myometrium, two-layer closure - inner myometrial layer + outer reinforcing layer (most frequently used, three-layer closure - used in deep or large defects for additional strength, serosal closure - final outer layer to reduce adhesions and improve healing, and endometrial cavity repair (if opened) - meticulous closure to restore uterine anatomy [4,5]. The increasing use of laparoscopic myomectomy has raised concerns regarding scar integrity due to technical limitations, such as restricted tactile feedback, and challenges in achieving multilayer myometrial closure [5]. While systematic reviews have not consistently demonstrated a statistically significant difference in rupture risk between laparoscopic and open approaches, careful surgical technique remains critical [2,6].

## Case presentation

A 36-year-old G3P0 woman at 28 weeks and 5 days of gestation was brought to the maternity emergency department by ambulance due to the sudden onset of severe abdominal pain and dizziness. The patient was hemodynamically unstable on presentation, as reflected by her clinical condition, as evidenced by hypotension and tachycardia: *tensio arterialis* or arterial pressure (T/A): 80/50 mmHg, heart rate (HR): 116 bpm, respiratory rate (RR): 22, oxygen saturation (SpO2): 97%, and temperature (T): 36.7 °C, which prompted immediate concern for an acute intra-abdominal event and the decision to proceed with emergency laparotomy. We have clarified this statement in the revised manuscript for better consistency. The pain had begun abruptly approximately 30 minutes prior to admission, was diffuse and intense in nature, and was accompanied by generalized weakness and dizziness. Given the rapid progression of symptoms, emergency medical services were activated.

Her obstetric history was significant for a prior left tubal ectopic pregnancy in 2020, treated with laparoscopic salpingectomy, followed by a spontaneous miscarriage at four to five weeks of gestation in 2023 with no need for further intervention. Her gynecological history was notable for multiple uterine leiomyomas located in the posterior wall of the uterus, for which she had undergone laparoscopic myomectomy in 2020. The postoperative course had been complicated by intra-abdominal hemorrhage and sepsis requiring repeat laparoscopic intervention. Despite our efforts, we were unable to obtain surgical records from the prior procedure.

The current pregnancy had been monitored with regular antenatal care and was classified as high risk. During the course of the pregnancy, the patient experienced episodes of threatened miscarriage and threatened preterm labor. At 24 weeks of gestation, a cervical pessary was placed due to cervical insufficiency. Two weeks prior to the current presentation, she had been hospitalized for threatened preterm labor and received a complete course of antenatal corticosteroids for fetal lung maturation. In addition, she was receiving low-molecular-weight heparin for thrombophilia.

**Figure 1:**
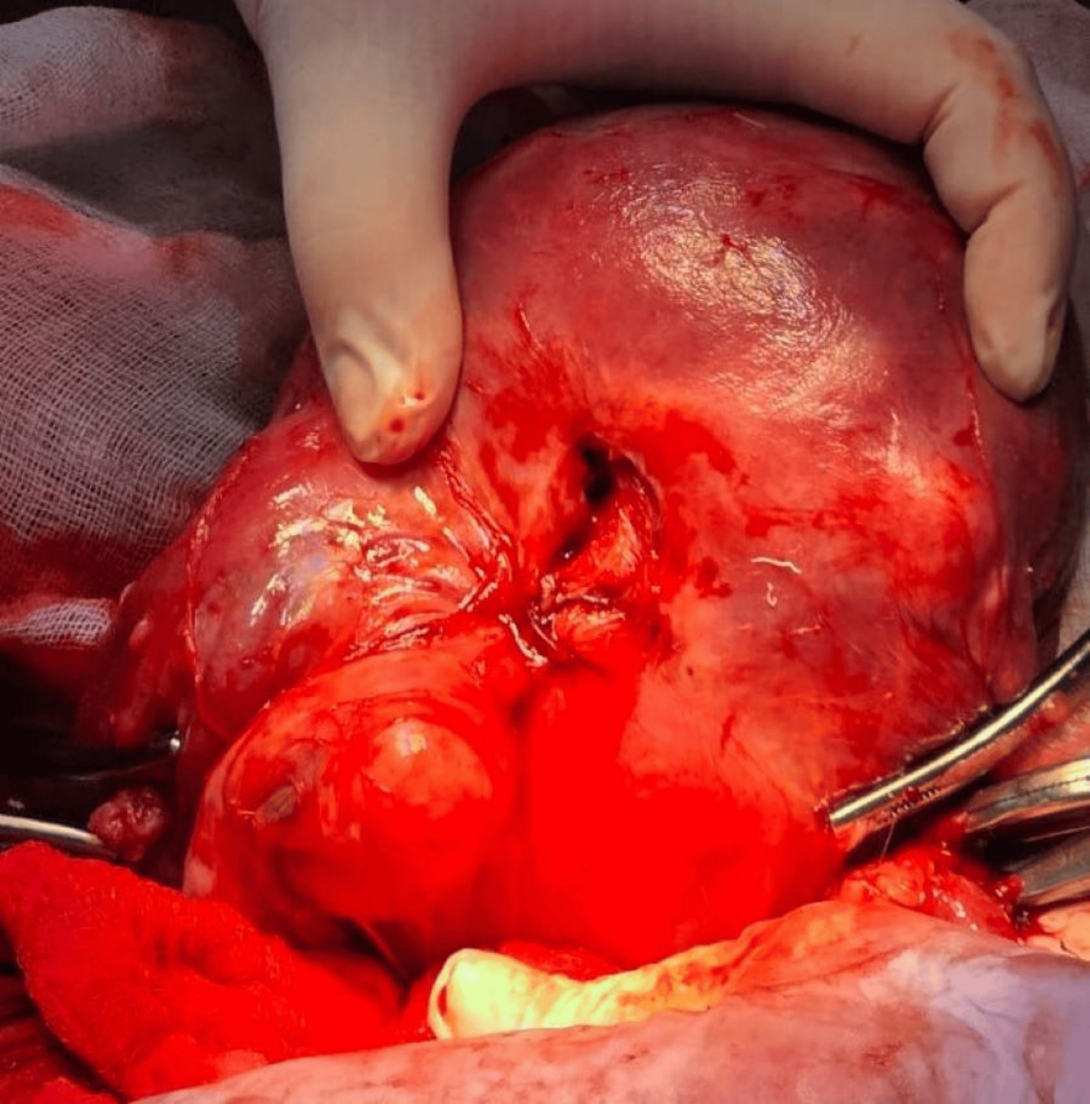
Intraoperative view of posterior uterine rupture with active hemorrhage Intraoperative image demonstrating a defect in the posterior wall of the lower uterine segment with active bleeding. A temporary hemostatic suture was placed to control hemorrhage prior to definitive surgical management.

On presentation, the patient was hemodynamically unstable, raising immediate concern for an acute intra-abdominal process. An emergency laparotomy was performed. Intraoperative findings revealed approximately 1000 mL of hemoperitoneum consisting of both liquid blood and clots. Inspection of the uterus demonstrated a 3 cm rupture located in the posterior wall of the lower uterine segment with active bleeding (Figure 1). The uterus was noted to be significantly distorted by multiple intramural leiomyomas. Examination of the adnexa revealed the absence of the right ovary, while the left ovary was fibrotically degenerated, enlarged, and densely adherent to the posterior uterine wall. The left fallopian tube was also absent.

Following evacuation of the hemoperitoneum, a lower uterine segment incision was performed, and a live preterm female neonate was delivered in cephalic presentation. The neonate weighed 1200 g, measured 37 cm in length, and had Apgar scores of 5 and 6 at 1 and 5 minutes, respectively. The neonate was immediately transferred to the neonatal intensive care unit for further management.

After delivery of the placenta, intraoperative reassessment confirmed the posterior uterine rupture and extensive distortion of the uterine anatomy due to multiple fibroids. In the setting of ongoing hemorrhage, significant uterine pathology, and a complex prior surgical history, the decision was made to proceed with total hysterectomy. The procedure included adhesiolysis, ligation of uterine vessels, division of the cardinal and uterosacral ligaments, and removal of the uterus and cervix, along with partial resection of the left ovary. A drain was placed in the Douglas pouch, and the abdomen was closed in layers. The total estimated blood loss was approximately 1500 mL, and the patient received 1 unit of packed red blood cells intraoperatively.

The postoperative course was uneventful. The patient required an additional blood transfusion for post-hemorrhagic anemia but remained hemodynamically stable throughout her recovery. No infectious or surgical complications were observed, and she was discharged home in satisfactory condition. The neonate remained in the neonatal intensive care unit and required mechanical ventilation; however, gradual improvement in respiratory function was noted during the early postnatal period.

## Discussion

Uterine rupture after conservative myomectomy is an uncommon but potentially catastrophic complication of pregnancy. Published systematic reviews and meta-analyses estimate the overall risk of uterine rupture following myomectomy to be approximately 0.5-1%, although the true incidence may vary depending on surgical technique, fibroid characteristics, and individual patient factors [1,2]. Most reported cases occur in the late second or third trimester, often before the onset of labor, and may present with acute abdominal pain, intra-abdominal hemorrhage, fetal distress, or sudden maternal hemodynamic instability [3,4].

Several factors have been suggested to influence the risk of uterine rupture following myomectomy. These include the number, size, and location of fibroids, the depth of myometrial incision, and the method of uterine wall reconstruction. Extensive intramural dissection and inadequate approximation of myometrial layers may potentially weaken the uterine scar, predisposing it to rupture during the progressive distension of pregnancy [4,5].

In recent decades, laparoscopic myomectomy has increasingly replaced traditional open surgery due to its well-established perioperative advantages, including reduced intraoperative blood loss, shorter hospital stay, faster postoperative recovery, and potentially lower adhesion formation. Despite these benefits, concerns have been raised regarding the integrity of the uterine scar following laparoscopic procedures. Some authors have suggested that the use of electrosurgical energy, limited tactile feedback, and technical challenges in performing multilayer myometrial closure laparoscopically may theoretically affect scar strength [7-9]. However, the available literature remains inconclusive. Several systematic reviews and pooled analyses have not demonstrated a statistically significant difference in uterine rupture rates between laparoscopic and open myomectomy, although a non-significant trend toward slightly higher rupture rates following laparoscopic procedures has been reported in some observational studies [8].

Within the clinical practice of the Perinatology Department of the First University Clinic of Tbilisi State Medical University (TSMU), a tertiary referral center for high-risk pregnancies, we noted that uterine rupture in patients with prior myomectomy appears to occur more frequently in women with a history of laparoscopic conservative myomectomy than in those who previously underwent open (laparotomic) surgery. It is important to emphasize that this represents a clinical impression based on institutional experience rather than findings from a formally designed clinical study, and therefore, it should not be interpreted as evidence of a causal association.

Nevertheless, this observation is consistent with ongoing discussions in the literature emphasizing the potential importance of surgical technique and the quality of uterine wall reconstruction in determining long-term scar integrity. Open myomectomy may provide more direct visualization and tactile feedback, facilitating careful multilayer closure of the myometrium with delayed-absorbable sutures. In contrast, laparoscopic repair may, in some cases, be technically more challenging, particularly when large or deeply intramural fibroids are removed.

Given the increasing number of reproductive-aged women undergoing minimally invasive myomectomy, further research is necessary to clarify whether the surgical approach itself influences the risk of uterine rupture or whether fibroid characteristics, surgeon experience, and closure technique primarily determine outcomes. Prospective multicenter studies with standardized documentation of surgical techniques, including the number of uterine incisions and methods of myometrial closure, would be particularly valuable in addressing this question [10-12].

Uterine rupture is known to occur more frequently in patients who have undergone multiple myomectomies, likely due to weakened myometrial integrity. Additionally, pregnancies achieved through in vitro fertilization (IVF) may carry an increased risk of obstetric complications, including preterm labor and abnormal placentation [13]. Therefore, early detection and vigilant monitoring are crucial to optimize both maternal and fetal outcomes.

The present case underscores the importance of maintaining a high index of suspicion for uterine rupture in pregnant patients with a history of uterine surgery who present with sudden abdominal pain, even in the absence of labor. Early recognition and prompt surgical intervention remain essential for optimizing maternal and neonatal outcomes.

## Conclusions

Uterine rupture after conservative myomectomy is a rare but serious complication that can occur in the late second or third trimester of pregnancy. This case emphasizes the importance of considering pregnancies after myomectomy as high-risk, maintaining vigilance for acute abdominal symptoms, and ensuring prompt surgical intervention when rupture is suspected. Observations from our tertiary referral center suggest that careful surgical technique and quality of uterine reconstruction may influence long-term scar integrity, highlighting the need for standardized approaches and further research. Clinicians should remain alert to these risks and manage such patients with close monitoring throughout pregnancy. Patients with a history of laparoscopic conservative myomectomy should be considered at increased risk for uterine rupture during pregnancy, particularly when the extent of myometrial dissection or closure technique is unknown. Therefore, enhanced antenatal surveillance is recommended. Although there is no universally standardized protocol, most authors and guidelines suggest more frequent antenatal visits compared to low-risk pregnancies, with particular attention during the second and third trimesters. Ultrasound assessment plays an important role in the follow-up of these patients. Serial sonographic evaluation of the uterine wall and scar region may help identify areas of significant thinning, although the predictive value of scar thickness for uterine rupture remains uncertain. In selected cases, magnetic resonance imaging (MRI) may provide additional information regarding myometrial integrity. Clinical management should include detailed patient counseling regarding warning symptoms such as persistent abdominal pain, uterine tenderness, or signs of intra-abdominal bleeding. Patients should be advised to seek immediate medical attention if such symptoms occur. Physical activity may be moderated on an individual basis, although strict activity restriction is not routinely recommended in the absence of symptoms. Delivery planning is crucial. Elective cesarean section prior to the onset of labor is often recommended, especially in cases with multiple myometrial incisions, deep intramural fibroids, or unclear surgical details. Timing of delivery is typically individualized, with many clinicians favoring delivery at 36-38 weeks to minimize the risk of spontaneous labor. Overall, a multidisciplinary approach and individualized care plan are essential to optimize maternal and fetal outcomes in pregnancies following laparoscopic myomectomy.
